# Graphene Oxide Improves *in vitro* Fertilization in Mice With No Impact on Embryo Development and Preserves the Membrane Microdomains Architecture

**DOI:** 10.3389/fbioe.2020.00629

**Published:** 2020-06-16

**Authors:** Nicola Bernabò, Luca Valbonetti, Marcello Raspa, Antonella Fontana, Paola Palestini, Laura Botto, Renata Paoletti, Martin Fray, Susan Allen, Juliana Machado-Simoes, Marina Ramal-Sanchez, Serena Pilato, Ferdinando Scavizzi, Barbara Barboni

**Affiliations:** ^1^Faculty of Bioscience and Technology for Food, Agriculture and Environment, University of Teramo, Teramo, Italy; ^2^National Research Council – Institute of Biochemistry and Cell Biology, Rome, Italy; ^3^Department of Pharmacy, D’Annunzio University of Chieti–Pescara, Chieti, Italy; ^4^School of Medicine and Surgery, University of Milano-Bicocca, Monza, Italy; ^5^Allevamenti Plaisant srl, Rome, Italy; ^6^MRC-Harwell, Oxford, United Kingdom

**Keywords:** mouse spermatozoa, graphene oxide, sperm capacitation, cholesterol, *in vitro* fertilization, sperm membrane, rafts, detergent resistant membrane

## Abstract

During the latest years, human infertility worsened all over the world and is nowadays reputed as a global public health issue. As a consequence, the adoption of Assisted Reproductive Technologies (ARTs) such as *In Vitro* Fertilization (IVF) is undergoing an impressive increase. In this context, one of the most promising strategies is the innovative adoption of extra-physiological materials for advanced sperm preparation methods. Here, by using a murine model, the addition of Graphene Oxide (GO) at a specific concentration has demonstrated to increase the spermatozoa fertilizing ability in an IVF assay, finding that 0.5 μg/ml GO addition to sperm suspensions before IVF is able to increase both the number of fertilized oocytes and embryos created with a healthy offspring given by Embryo Transplantation (ET). In addition, GO treatment has been found more effective than that carried out with methyl-β-cyclodextrin, which represents the gold standard in promoting *in vitro* fertility of mice spermatozoa. Subsequent biochemical characterization of its interaction with male gametes has been additionally performed. As a result, it was found that GO exerts its positive effect by extracting cholesterol from membranes, without affecting the integrity of microdomains and thus preserving the sperm functions. In conclusion, GO improves IVF outcomes *in vitro* and *in vivo*, defining new perspectives for innovative strategies in the treatment of human infertility.

## Introduction

In the recent years, our society has witnessed a concerning decay of human fertility, as claimed by the WHO (Mascarenhas et al., 2012) and the European IVF-Monitoring Consortium (EIM), group of the European Society of Human Reproduction and Embryology (ESHRE) (De Geyter et al., 2018). From a clinical perspective, infertility is defined as the inability of a couple to achieve a pregnancy over 12 months of regular and unprotected sexual intercourse (Barratt et al., 2017). This medical condition affects approximately 15–20% (48.5 million) of the couples worldwide (Mascarenhas et al., 2012). In parallel, the use of Assisted Reproduction Technology (ART) as a specific therapeutic strategy has been characterized by an impressive increase since 1978 with the birth of Louise Brown, the first baby conceived by *in vitro* fertilization (IVF) in the world and, more recently, thanks to the awarded of Nobel prize in Physiology or Medicine for the development of Human *In Vitro* Fertilization (Edwards, 2001).

A successful fertilization can be achieved by the ICSI (Intra-Cytoplasmic Sperm Injection) process, an invasive technique in which a spermatozoon is directly injected into an oocyte or by IVF, a more physiological technology in which spermatozoa are free allowed to recognize, interact with and fertilize matured oocytes.

In accordance with the EIM, gathering data at the present time from 1343 European clinics, the number of cycles performed has incredibly raised up to 849,811 in 2015 (+9.4% than in 2014) in Europe, with a total of 187,542 infants born after ART (De Geyter et al., 2020). It is important to mind that, as reported by the Grand View Research Incorporation^1^ “the global assisted reproductive technology (ART) market is expected to reach USD 45.4 billion by 2025.”

To date, IVF outcomes are still far from optimal and with a relatively low and shifting efficiency (25–95%) (Byers et al., 2006) with possible negative effects on embryo quality (Hu et al., 1998; Strandell et al., 2000; Katayama et al., 2010; Duranthon and Chavatte-Palmer, 2018; Ramos-Ibeas et al., 2019). For these reasons, researchers are constantly seeking novel approaches to enhance ART and are always prone to adopt different materials and methods.

In this context, medical research benefits enormously from experiments carried out using animal models, of which the laboratory mouse (*Mus musculus*) being widely recognized as essential for the advancement of science and translational medicine (Matthews, 2008; Barré-Sinoussi and Montagutelli, 2015). Mice are the most commonly used animal model for studying human diseases, since they are biologically and evolutionarily similar to humans and suffer from many of the same symptoms and diseases (Barré-Sinoussi and Montagutelli, 2015; Perlman, 2016). Overall, it is worth to notice that mice and humans share virtually the same set of genes and a common ancestor approximately 80 million years ago^2^ ^,3^ (accessed on 13/01/2020). Almost every gene found in one species so far has been found in a closely related form in the other. Both the mouse and human genomes contain about 3.1 billion base pairs and, on average, the protein-coding regions of the mouse and human genomes are 85 percent identical. Therefore, the genomes of all mammals are comparably similar, whilst mouse and human genomes are the most studied ones and the first produced and analyzed (HGP: 2000; MGP: 2002).

In this research project, we explore the use of graphene oxide (GO) as a possible material to refine *in vitro* fertility and get healthy newborns. Graphene is a thin, two-dimensional layer of carbon atoms arranged in a hexagonal lattice. Known as a “wonder material” graphene has unique mechanical, thermal, electrical and optical properties (Zhu et al., 2010; El Achaby et al., 2012; Novoselov et al., 2012). Due to its wide range of applications, molecular interactions between graphene and derivatives with cell membranes has drawn the attention of researchers, especially neuroscientists. It is interesting to notice that graphene based-substrates have been studied as a support for neuronal functional development, retaining unaltered the neuronal signaling properties (Fabbro et al., 2016). Later on, Rauti et al. (2016) demonstrated that the treatment with GO flakes down-regulated the neuronal signaling without affecting cell viability. Contemporaneously, another work demonstrated the interactions between GO and the cell membrane, with a special focus on lipid modifications derived from this interaction. More in detail, the authors revealed an upregulation of phosphatidylethanolamines (PEs) and a downregulation of phosphatidylserines (PSs) in the plasma membrane of neurons and synaptic vesicles after the treatment with GO flakes, changing the PE/PS ratio and thus the lipids content. Surprisingly, even if the GO exposure showed some effects on neuronal transmission and network functionality, this interaction did not affect the cell viability and network formation (Bramini et al., 2016).

Recently, a significant improvement has been found in fertility when sperm were exposed to GO during the process that leads them to become fertile, the capacitation, as recently published in swine (Bernabò et al., 2018) and bovine models (Ramal-Sanchez et al., 2019), probably due to an extraction of cholesterol from the sperm membrane and thus an intense lipid membrane remodeling (Bernabò et al., 2019).

Here, we carried out a set of experiments in order to explore the effects of GO on murine sperm capacitation. Micrometric sheets of graphene, oxidized by a patented modification of the Hummers’ method (Hummers and Offeman, 1958; Zurutuza Elorza and Alonso Rodriguez, 2016) in order to obtain an oxygen percentage of 41–50% (see product datasheet) were added to sperm incubation media, then trials of IVFs and ETs were performed.

As the main conclusion, we found that the co-incubation of capacitating spermatozoa with a specific concentration of GO (0.5 μg/ml) was able to increase the number of both fertilized oocytes and developed embryos/birth rates without exerting toxic collateral effects. Then, GO has been also positively used in combination with methyl-β-cyclodextrin (MβCD) which is reputed the gold standard method for improving sperm capacitation in murine IVF protocols. Lastly, we proposed a mechanistic model of the GO-sperm membrane interaction, in which GO extracts cholesterol from Liquid disordered phase of membranes, without affecting the liquid ordered domains.

## Materials and Methods

### Chemicals

Graphene oxide, GO, was a commercial sample from Graphenea, Donostia-San Sebastian (Spain), prepared using a patented modification of the Hummers’ method (Hummers and Offeman, 1958; Zurutuza Elorza and Alonso Rodriguez, 2016). The product datasheet confirmed the following elemental analysis: 49–56% C, 0–1% H, 0–1% N, 2–4% S, 41–50% O. In particular XPS analyses performed by Graphenea evidenced the presence of C-C, C-O, C = O and O = C-O moieties.

All the chemicals were purchased from Sigma Aldrich (Saint Louis, MO, United States) and were analytical grade. Human tubal fluid (hTF) was purchased from Millipore (Merck, KGaA, Darmstadt, Germany) and modified with the addition of CaCl_2_ to increase the Ca^2+^ concentration from 2.04 mM (regular concentration) to 5.14 mM (high concentration) (Li and Glass, 2016). For sperm capacitation, TYH medium (containing 1 mg/ml PVA) was prepared in-house (Choi and Toyoda, 1998).

The protease inhibitor cocktail used was from Roche (Switzerland). Primary antibody against Caveolin was obtained from BD Transduction Laboratories (San Jose, CA, United States) and the primary antibody against CD55 was from Santa Cruz Biotechnology (CA, United States). The secondary antibody used for enhanced chemiluminescence (ECL) detection was an anti-rabbit HRP conjugate from Pierce (Rockford, IL, United States). All material for electrophoresis were purchased from Bio-Rad (Milan, Italy).

The reagents used (analytical grade) and HPTLC plates (Kieselgel 60), for lipid analysis, were purchased from Merck KGaA (Darmstadt, Germany).

### Graphene Oxide Characterization

#### Aqueous Dispersion of GO and Characterization

The commercial aqueous solution of GO was diluted at the elected concentration and bath ultrasonicated for 10 min (Elmasonic P60H, 37 kHz, 180W). Reagent sterilization was performed by irradiation under UV lamp (Spectronics Spectroline EF 160/C FE, 6W, 50 Hz, 0.17 A) for 2 h. The final concentration of GO was checked by UV-vis spectrophotometry (Varian Cary 100 BIO) at λmax 230 nm. The size of GO sheets was measured in water at 25 and 38.5°C by using Dynamic Laser Light Scattering (90Plus/BI-MAS ZetaPlus multi-angle particle size analyzer, Brookhaven Instruments Corp.).

### Testing of GO Effects on IVF Outcomes

#### Mice and Husbandry

All animal experiments described in this paper were bred at the National Research Council-Institute of Biochemistry and Cell Biology *(CNR-IBBC)*, Infrafrontier-European Mouse Mutant Archive (EMMA), Specific Pathogen-Free (SPF) barrier unit (Monterotondo Scalo, Rome, Italy). Mice were housed in Individually Ventilated Caging Systems (Tecniplast, Gazzada, Italy) at a Temperature (T) of 21 ± 2°C, Relative Humidity (RH) of 55 ± 15% with 50–70 Air Changes per Hour (ACH) and under controlled (12:12 h) light–dark cycle (7 am–7 pm). Mice had *ad libitum* access to water and a standard rodent diet (Emma 23, Mucedola, Settimo Milanese, Italy). Spermatozoa were frozen from C57BL/6NCnrm males of 3 months of age. The C57BL/6NCnrm females used as oocyte donors for the IVF were of 3–4 weeks of age. The mice were culled by trained personnel using gaseous anesthesia followed by a rising concentration of CO_2_ and cervical dislocation to confirm death or cervical dislocation alone. All the experimental procedures were agreed upon, reviewed and approved by local animals welfare oversight bodies (CNR), the experiments were performed with the approval and direct supervision of the CNR-IBCN/Infrafrontier—Animal Welfare and Ethical Review Body (AWERB), in accordance with general guidelines regarding animal experimentation, approved by the Italian Ministry of Health, in compliance with the Legislative Decree 26/2014 (ref. *Project license A80EE.N.7KR;* Project license PPL30/3358), transposing the 2010/63/EU Directive on protection of animals used in research. This work was also conducted using recommendations taken from the both ARRIVE and PREPARE guidelines (Kilkenny et al., 2010; Smith et al., 2018).

#### Sperm Cryopreservation

Sperm cryopreservation was performed using the protocol described by Ostermeier et al. (2011). The cauda epididymis and the vasa deferentia from 3 males were pooled in a sperm collection dish containing 3 ml cryoprotective medium (CPM): 18% w/v raffinose (Sigma Aldrich, cat #R7630), 3% w/v skim milk (BD Diagnostics, cat # 232100) and 477 μM monothioglycerol (Sigma Aldrich, cat # M6145). Spermatozoa were released into the CPM for 10 min at 37°C, then the pooled sperm was loaded into 0.25 ml French straws (IMV Technologies, France), each containing three aliquots of approximately 12 μl each. Straws were placed onto a polystyrene raft floating on LN_2_ for 10 min before being plunged into LN_2_ and stored in a LN_2_-tank until use.

#### GO Dispersion for *in vitro* Analyses

In the present study, solutions at different concentration of GO were used, at respectively: 50, 10, 5, 1, 0.5, and 0.1 μg/ml. These concentrations were chosen according to our previous works (Bernabò et al., 2018; Ramal-Sanchez et al., 2019). GO was solubilized in TYH medium (NaCl 119.37 mM; KCl 4.78 mM; CaCl_2_×2H_2_O 1.71 mM; KH_2_PO_4_ 1.19 mM; MgSO_4_×7H_2_O 1.19 mM; NaHCO_3_ 25.07 mM; Na pyruvate 1 mM; Glucose 5.56 mM; PVA 1 mg/ml; Penicillin G potassium 7.5 mg/100 ml; Streptomycin sulfate 5 mg/100 ml). The solutions were freshly prepared for each experiment.

#### *In vitro* Fertilization (IVF)

To evaluate the effect of the graphene oxide (GO) on mouse frozen spermatozoa, a series of IVF experiments were performed, using the different concentrations of GO (see above).

The IVF’s were set up using the *in vitro* fertilization method described by Ishizuka et al. (2014). Briefly, the oocytes were obtained from C57BL/6NCnrm females at 3–4 weeks of age. Females were induced to super ovulate by intraperitoneal injection of 5 IU PMSG (Intervet, Milan, Italy), followed by 5IU hCG (Intervet) 48 h later. For each IVF session two frozen straws were rapidly thawed by transferring them into a 37°C water bath for 8 min. The two straws were considered two experimental groups. Thawed spermatozoa from one straw were added into 90 μl pre-equilibrated TYH (control group), while thawed spermatozoa from the other straw were added into 90 μl pre-equilibrated TYH with GO (treated group). To enhance sperm capacitation and enrich progressive motility spermatozoa were incubated for 30 min at 37°C under 5% CO_2_.

Twenty minutes before insemination, cumulus oocyte complexes (COCs) from females were released into a 250 μl fertilization drop, consisting of hTF medium (Merck Millipore cat # MR-070-D) with 1 mM GSH (Sigma Aldrich, cat #G4251) and incubated. To reduce the female variability, one cumulus oocyte complex (COC) from each female was used for the control IVF dish and the other for the treated IVF dish. For each IVF session, COCs from a total of 12 females were divided into 6 fertilization dishes, 3 dishes for the control group, and 3 dishes for the treated group.

After incubation, 20 μl of sperm were collected from the peripheral part of each capacitation drop and transferred to the fertilization dish (spermatozoa final concentration 2 to 6 × 10^5^ cells/ml).

After 4 h, the inseminated oocytes were washed three times in drops of 100 μL hTF and were then cultured at 37°C under 5% CO_2_. Twenty four hours after insemination, the fertilization rates were calculated as percentage of the total number of two-cell embryos obtained divided by the total number of inseminated oocytes. In total, for each GO concentration examined, at least three replicates were performed and a total number of 2632 oocytes were used.

#### Assessment of GO Effect on Spermatozoa Acrosome Integrity

As previously described, the acrosome integrity of spermatozoa was monitored by using a two-staining technique with Hoechst 33258 and FITC-PSA able to identify alive unreacted and reacted spermatozoa (Mattioli et al., 1996). At least 100 cells have been assessed by fluorescence microscopy in three independent experiments performed at different capacitation times on control (CTRL) and GO exposed spermatozoa.

### Assessment of Potential Toxic Effects Derived From GO Exposure of Capacitating Spermatozoa on Early Embryo Development and Birth Rate

#### Embryo Quality Control (QC)

For each treatment, 10 embryos were randomly collected and fixed in PBS + 4% paraformaldehyde for 1 h, then stored at 4°C in PBS for morphological analysis. To this aim, they were stained with DilC12 (for membrane staining) and DAPI (for nuclear staining), then examined under confocal microscopy (Nikon A1R) for normal embryo morphology.

From a functional point of view, the quality of the embryos generated by IVF was assessed *in vitro* by culturing IVF derived 2-cell embryos in potassium simplex optimized medium supplemented with amino acids (KSOM^AA^; Ho et al., 1995) for 72 h until reaching the blastocyst stage and recording the development rate achieved. Results are expressed as a percentage of blastocysts per 2-cell embryos cultured.

#### Embryo Transplantation (ET)

IVF derived embryos generated using sperm exposed to 0.5 μg/ml GO were frozen down at the 2-cell stage using a method previously described (Infrafrontier-EMMA, European Mouse Mutant Archive^4^, accessed on 13/01/2020). This was done to accumulate a pool of embryos that could be transferred into recipient females as part of the same experiment. CD-1 females were used as embryo recipients on day 0.5 of pseudopregnancy which was induced by mating to genetically sterile Prm1 males (Haueter et al., 2010). Sterile males were obtained from the MRC-MLC Infrafrontier/EMMA/FESA Repository (EM:12662). 114 control embryos and 98 embryos generated using GO-treated sperm were divided equally between 7 x pseudo-pregnant females/treatment group. All females were allowed to litter normally and the number of pups born was recorded along with the number of implantation sites observed at necropsy. This part of the work was conducted under project license PPL30/3358.

Embryo transfers were conducted under aseptic conditions using isoflurane anesthesia. Analgesia was provided by s.c. injection of buprenorphine (0.1 mg/kg) and butorphanol tartrate (1.0 mg/kg). The surgical ET was performed as previously described (Behringer et al., 2014).

### Evaluation of IVF Rates After Exposing Capacitating Spermatozoa to GO and/or MβCD

The IVF protocol was performed as previously described. Thawed spermatozoa were incubated for 30 min in a capacitation drop consisting of: TYH (control group), 0.5 μg/ml GO, 0.75 mM MβCD or GO and MβCD simultaneously. This experiment was repeated at least three times reaching a total number of 1659 oocytes. Results are referred to the percentage of oocytes that reached the 2-cell embryo stage.

### Biochemical Characterization of Mouse Sperm Membranes After Treatment With GO 0.5 μg/ml During Capacitation

#### Preparation of Triton Soluble (TS) and Triton Insoluble (TI) Fractions From Mouse Spermatozoa

The sperm pellet from 1000 × 10^6^ cells, according to Hyne and Garbers (1979), was resuspended in 1 ml of hypotonic buffer (2 mM Tris [pH 7.2], 12 mM NaCl) with a protease inhibitor cocktail and 1 mM PMSF. The cells were homogenized with 20 strokes in a tight-fitting glass Douce homogenizer.

Then, a membrane enriched fraction that included a fraction from endocellular membranes was prepared according to Preti et al. (1980). Briefly, the homogenates were centrifuged for 10 min at 1,000 *g* and, after recovering the supernatants, they were resuspended in 500 μL of a solution containing 1 mM Tris pH7.2, 320 mM sucrose, 0.1 mM EDTA, with a cocktail of protease inhibitors and 1 mM PMSF. The pellets were homogenized by pipetting up and down and centrifuged three times in the same buffer, and the pooled supernatants were centrifuged at 100,000 *g* for 1 h, 4°C. The pellet from the last centrifugation contained the membrane-enriched fraction (MEF).

Membrane-enriched fraction were utilized for membrane sub-fractionation. The membrane pellet was resuspended in MBS-buffered saline (MBS: 25 mM MES, pH 6.5, 150 mM NaCl) with a protease inhibitor cocktail and 1 mM PMSF. In order to maintain a constant protein/detergent ratio, a known volume containing 1 mg of protein was adjusted to 500 μL by mixing with MBS containing protease inhibitors. The whole procedure was carried out on ice to maintain the integrity of the lipid rafts. Then an equal volume of cold 2% Triton X-100 in MBS with protease inhibitors was added and mixed. Tubes were held on ice for 30 min. The cell lysate was centrifuged at 15,000 *g* for 30 min at 4°C to separate the supernatant (*triton soluble* fraction, TS) from the pellet (*triton insoluble* fraction, TI) containing lipid microdomains (Simons et al., 1999).

#### Western Blot Analysis

The protein content of TS and TI fractions was quantified by micro-BCA assay (Merck KGaA, Darmstadt, Germany). Thereafter, 15 μg of proteins were loaded onto a 12% polyacrylamide gel prior to SDS-PAGE analysis. Subsequently, proteins were transferred to membranes that were stained with Ponceau S to assess proper transfer. Blots were washed with TBS and blocked for 1 h in TBS-T/skimmed milk. After blocking, blots were incubated overnight with the primary antibody diluted in TBS-T/skimmed milk [anti-CAV-1 1:1,000, anti-CD55 1:200], and then for 1.5 h with HRP-conjugated anti-rabbit IgG (5,000-fold diluted in TBS-T/milk). TS and TI fractions were obtained from three independent experiments. Proteins were detected by ECL with the Super Signal detection kit (Thermo Scientific) and analyzed with ImageQuant^TM^ TL (GE Healthcare Life Sciences), program 1D gel analysis.

#### Lipid Analysis

Aliquots of TS and TI fractions were used for phospholipids phosphorus determination by the Bartlett procedure. Then, 7 μg of phosphorus from each sample were subjected to lipid extraction according to Tettamanti’s protocol (Tettamanti et al., 1973). The lipid extracts in the organic phases were analyzed by HPTLC. In particular, for phospholipid and cholesterol analysis the solvent system was chloroform/methanol/acetic acid/water (60/45/4/2, vol/vol/vol/vol). Phospholipids and cholesterol were visualized on the same HPTLC plate with anisaldehyde reagent (0.5 ml anisaldehyde, 1 ml 97% sulfuric acid in 50 ml glacial acetic acid). After heating the plate at 180°C for 5 min, the plates were scanned and analyzed with ImageQuant^TM^ TL (GE Healthcare Life Sciences), program 1D gel analysis.

### Statistical Analysis

All the data were checked for normal distribution with D’Agostino and Pearson normality test, then they were compared with parametric or non-parametric tests as required.

The curve fitting was obtained by using the best fitting technique, using linear or non-linear models.

In all the cases the differences among groups were considered statistically significant when *p* < 0.05, and statistically highly significant when *p* < 0.001.

To assess the effect of the sperm exposure to different GO concentrations (0.1, 0.5, 1, 5, 10, and 50 μg/ml) we carried out six independent biological experiments, performing two technical replicates each time, thus obtaining six pairs of values for each parameter (samples incubated in CTRL conditions vs. 5 GO concentrations). As a consequence, we analyzed the difference between the CTRL and treated samples, expressed as a percentage of the CTRL (Δ_IVF__%_).

## Results

### GO Dispersion Characterization

The dimensions of GO flakes in aqueous dispersions and at different concentrations were checked using Dynamic Light Scattering (3D LS Spectrometer) at 25 and 38.5°C. Samples measurements (Table 1) confirmed micrometric dimensions (i.e., size interval 600–900 nm). The polydispersity (interval 0.24–0.32) confirmed moreover the presence of not homogenous samples, comprising nano-objects of different dimensions, as expected. Dimensions anyway did not vary significantly upon increasing of the temperature, from room temperature to 38.5°C.

**TABLE 1:** Chemical characterization of GO solution at different concentrations.

GO concentration (μg/ml)	Size ± SD (nm)^a^ at 25°C	Polydispersity at 25°C	Size ± SD (nm)^a^ at 38.5°C	Polydispersity at 38.5°C
**0.5**	718 ± 110	0.287	720 ± 182	0.286
**1**	889 ± 145	0.322	842 ± 131	0.310
**5**	713 ± 76	0.290	641 ± 38	0.304
**10**	847 ± 306	0.292	629 ± 42	0.294
**50**	585 ± 14	0.241	607 ± 53	0.261

*^*a*^Mean of at least three measurements.*

### GO Affects IVF Outcomes in a Dose-Dependent Manner

#### GO Induce Acrosome Damage in a Dose-Dependent Manner

**FIGURE 1:**
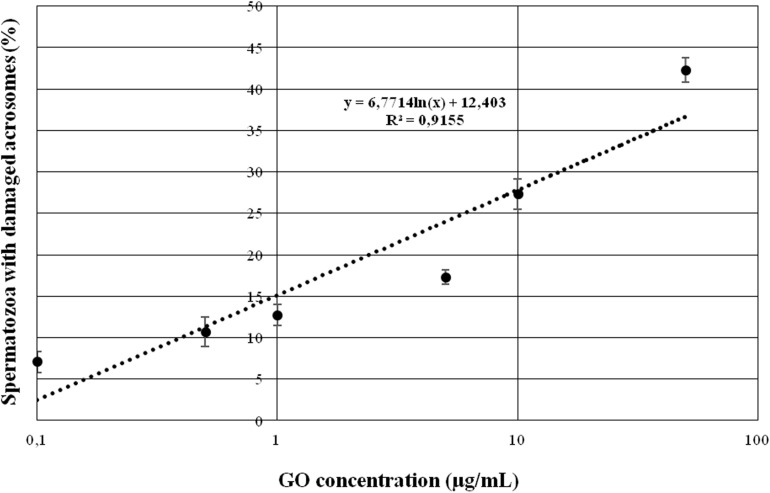
Graph showing the effects of increasing GO concentrations on sperm acrosome integrity when incubating under capacitating conditions.

As depicted in Figure 1, the treatment with GO was able to induce a statistically significant (*p* < 0.05) dose-dependent effect on acrosome integrity at concentrations higher than 1 μg/ml, whereas lower GO concentrations did not change the percentage of spermatozoa showing damaged acrosome when compared to the controls (*p* > 0.05).

#### GO 0.5 μg/ml Significantly Improves the IVF Outcomes

The effect of GO at different concentrations (0.1, 0.5, 1, 5, 10, and 50 μg/ml) on sperm incubation and on IVF outcome is reported in Table 2.

**TABLE 2:** Data summarizing the IVF outcomes when spermatozoa were incubated with different GO concentrations during capacitation, with respect to their controls.

Treatment	Mean ± SEM (%)	Treatment (μg/ml)	Mean ± SEM (%)	Δ_IVF_ (%)	*P*
**CTRL**	47.9 ± 5.0	**GO 0.1**	56.5 ± 4.6	17.9	0.276
**CTRL**	59.2 ± 5.2	**GO 0.5**	71.2 ± 3.8	20.1	**0.046**
**CTRL**	58.5 ± 7.2	**GO 1**	59.3 ± 3.3	1.3	0.925
**CTRL**	71.0 ± 6.4	**GO 5**	65.3 ± 4.9	−8.0	0.521
**CTRL**	62.4 ± 2.0	**GO 10**	58.4 ± 4.3	−6.4	0.450
**CTRL**	52.5 ± 2.7	**GO 50**	38.4 ± 1.0	−28.5	**0.008**

*Δ_IVF_% represents the difference in percentage of IVF outcomes between GO-treated and control sperms.*

**FIGURE 2:**
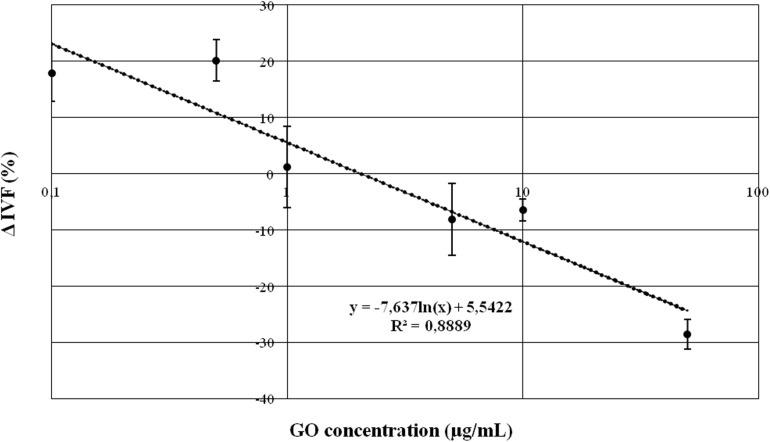
Graph representing the relationship between GO concentration and IVF outcomes, expressed as difference in percentage (Δ IVF%) respect to the CRTL.

Briefly, we found that none of the GO concentration we used exerted a statistically significant effect except the following ones: 0.5 μg/ml and 50 μg/ml. 0.5 μg/ml increased the fertilization rate by approximately 20% compared to the CTRL (*p* < 0.05), while the concentration of 50 μg/ml decreased the fertilization rate by approximately 30% when compared with the CTRL (*p* < 0.01). Figure 2 graphically shows the dose-dependency of GO concentration on IVF outcomes.

#### Spermatozoa Exposure to Different GO Concentrations Does Not Affect the Embryo Quality

**FIGURE 3:**
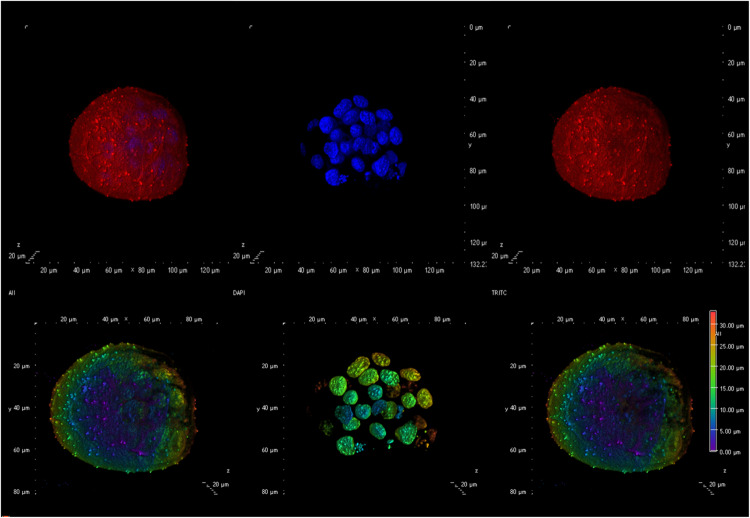
Confocal microscopy image showing an example of an embryo generated using spermatozoa exposed to 0.5 μg/ml GO. The **(upper panel)** shows an embryo with (from left to right) an overlay of the membranes in red (DilC12) and the nuclei in blue (DAPI), only the nuclei, only the membranes. The **(lower panel)** illustrates, from left to right an overlay of nuclei and membrane showing the Depth Coded maxIP (Intensity Projection) gradient visualization (to evaluate the 3D structure).

To assess the potential embryo-toxicity after sperm exposure to GO, quality of embryos generated was compared between GO-obtained embryos and CTRL embryos, as described in the previous paragraph. The morphological analysis did not reveal any detectable negative effect on embryos obtained from GO exposed spermatozoa (see Figure 3). The physiological analysis of the embryo development confirmed this finding, as summarized in Table 3.

**TABLE 3:** Data summarizing the embryo development to the blastocyst stage expressed as differences in percentage (Δ_Blasto_%) of embryo development in the CTRL conditions and embryo development of sperms exposed to different concentrations of GO.

Treatment	Mean ± SEM (%)	Treatment (μg/ml)	Mean ± SEM (%)	Δ_Blasto_ (%)	*P*
**CTRL**	95.0 ± 3.5	**GO 0.1**	100.0	5.3	ND
**CTRL**	95.0 ± 2.9	**GO 0.5**	97.5 ± 2.5	2.6	>0.05
**CTRL**	85.0 ± 5.0	**GO 1**	85.0 ± 5.0	0	>0.05
**CTRL**	90.0 ± 10.0	**GO 5**	100.0	11.1	ND
**CTRL**	95.0 ± 5.0	**GO 10**	95.0 ± 5.0	0	>0.05
**CTRL**	90.0 ± 5.0	**GO 50**	100.0	11.1	ND

In all the cases, statistical analysis showed that there were no significant differences between the development of embryos generated both in treated (different GO concentrations) and CTRL. It is noteworthy that we observed 100% development to the healthy blastocyst stage in embryos derived from GO treated spermatozoa. Furthermore, hatching rates were normal compared to the species feature.

#### Spermatozoa Exposure to Different GO Concentrations Does Not Affect the Birth Rate

**FIGURE 4:**
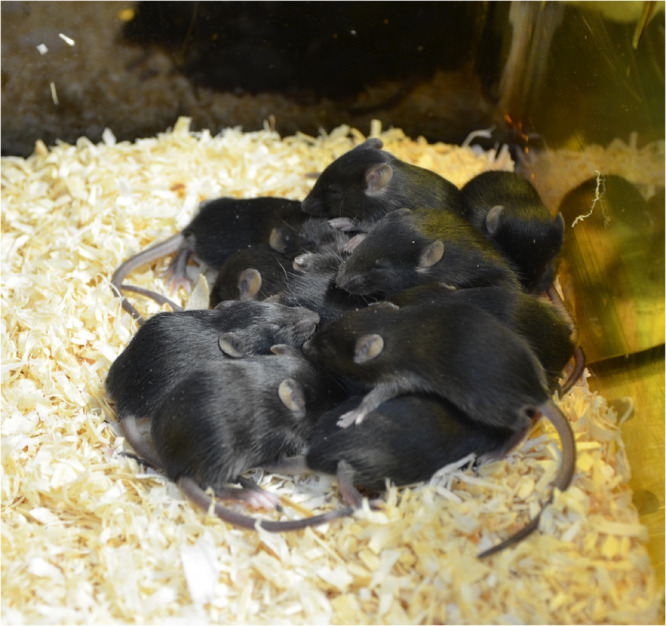
An example picture of pups derived by transferred embryos obtained after IVF with GO treated spermatozoa.

A total of 212 embryos were ET to pseudopregnant foster mothers (106 CTRL and 106 treated) and there was no difference in the number of implantation sites recorded between the CTRL (52.8%) and GO treatment group (59.5%), while the numbers of live born pups born was significantly and statistically different (CTRL 28/106 vs. GO 0.5 μg/ml 44/106, *p* = 0.02, χ^2^ Test). Two of the females receiving the CTRL embryos failed to litter successfully, but an example of the healthy-derived pups can be observed in Figure 4.

### Comparison of the Effect on IVF Exerted by the Exposure of Capacitating Spermatozoa to GO and/or Methyl-β-Cyclodextrin (MβCD)

#### GO 0.5 μg/ml and MβCD 0.75 mM Exert an Additive Effect on Sperm Capacitation When Used Simultaneously

As it is evident in Table 4, the two molecules seem to have a significant additive effect (*p* < 0.001).

**TABLE 4:** Data summarizing the IVF outcome, expressed as the difference in percentage change (ΔIVF%) of the fertilization rates in experiments carried out with spermatozoa exposed to GO (0.5 μg/ml) and/or MβCD 0.75 mM respect to the CRTL.

CTRL	GO 0.5 μg/ml	MβCD 0.75 mM	GO 0.5 μg/ml + MβCD 0.75 mM
57.9 ± 3.1	73.5 ± 1.0	70.3 ± 1.3	81.0 ± 0.6
**Δ_IVF_ (%)**	26.9	21.4	39.9
***P***	0.003	0.01	0.0004

### Biochemical Characterization of GO 0.5 μg/ml on Mouse Capacitating Sperm Membrane

#### Cav-1 Is Strongly Abundant in TS Fraction of Sperm Membranes

**FIGURE 5:**
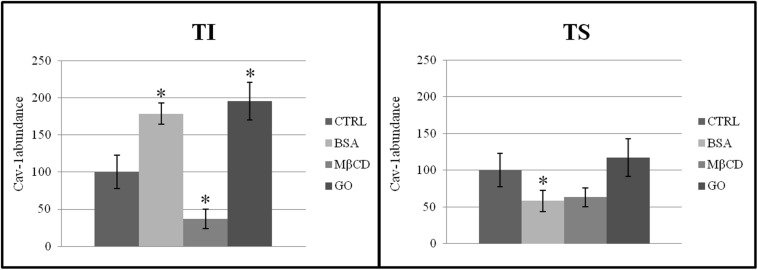
Distribution of Cav-1 between insoluble (TI) and soluble (TS) membrane fractions, isolated from MEF prepared from sperm incubated in a capacitating medium (BSA, MB CD, GO treatments), or from sperm collected in a non-capacitating medium (CTRL). The data reported were expressed as cav-1 abundance in each group. Values are means ± SE for three separate experiments. *P < 0.05 versus control.

To explore the effect of GO on membrane chemical composition and architecture, two proteins known to be enriched in lipid microdomains were evaluated: Cav-1 (marker of *caveolae*) and CD55 (marker of lipid rafts). TS and TI fractions, probed with anti-Caveolin, revealed the presence of Cav-1 strongly in TS. The amount of Cav-1 increased significantly in TI after sperm capacitation with BSA and GO, but not with MβCD. Moreover, Cav-1 decreased in TS after BSA treatment (Figure 5).

**FIGURE 6:**
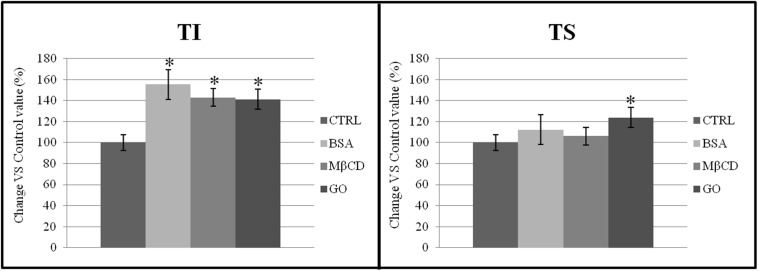
Percentage distribution of proteins in TI and TS fractions, isolated from a membrane-enriched fraction (MEF) prepared from sperm incubated in a capacitating medium (BSA, MβCD, GO treatments) or from sperm collected in a non-capacitating medium (CTRL). The data reported were expressed as percentage change relative to control value. Values are the mean of three protein determinations ± SE obtained from three independent experiments. *P < 0.05 versus control.

The presence of CD55 exclusively in the TI fraction of both non-capacitated and capacitated samples confirmed the purification of TI had been performed correctly (data not shown). As shown in Figure 6, the total membrane protein content increased significantly in TI after capacitation when treated with BSA, MβCD and GO. Moreover, the total amount of membrane protein also increased significantly in TS but only after treatment with GO.

#### BSA, MβCD and GO Reduce the Cholesterol and Phospholipids Ratio

**FIGURE 7:**
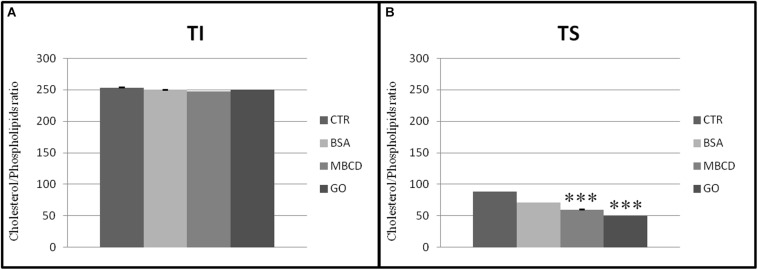
Cholesterol/Phospholipids ratio in **(A)** insoluble (TI) and **(B)** soluble (TS) membrane fractions, isolated from MEF prepared from sperm incubated in a capacitating medium (BSA, MβCD, GO treatments) or from sperm collected in a non-capacitating medium (CTRL). Values are means ± SE for three separate experiments. ***p < 0.001 versus control.

**FIGURE 8:**
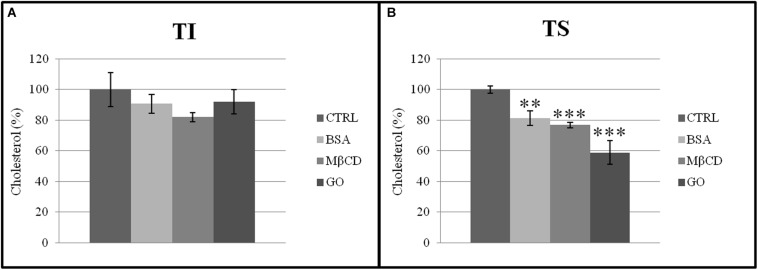
Cholesterol normalized to total protein content in **(A)** insoluble (TI) and **(B)** soluble (TS) membrane fractions, isolated from MEF prepared from sperm incubated in a capacitating medium (BSA, MβCD, GO treatments), or from sperm collected in a non-capacitating medium (CTRL). Values were expressed as percentage change relative to control value. Values are means ± SE for three separate experiments. **p < 0.01 versus control, ***p < 0.001 versus control.

The presence of acceptors (BSA, MβCD, and GO) in the capacitation buffer caused a reduction of the cholesterol and phospholipid ratio, promoting the extraction of cholesterol from both TS and TI fractions (Figure 7). However, the cholesterol content only decreased significantly in the TS fractions, especially after GO treatment (Figure 8).

## Discussion

Innovative strategies leading to the improvement of ARTs performances in general, and of IVFs in particular, have attracted the attention of the scientific community in the last decades. In this context, the use of new materials and methods on animal models may offer important findings to be readily translated to Human (Guan et al., 2014). This can maximize beyond the effect of personalized cures. Our group recently demonstrated the beneficial effects on IVF outcomes (Bernabò et al., 2018; Ramal-Sanchez et al., 2019) of co-incubating boar and bull spermatozoa with a suspension of GO. Therefore, in the present work we carried out a series of experiments aimed to investigate GO effect on embryo development and birth rate as well as to elucidate the mechanism of interaction between GO and sperm membrane during capacitation. Indeed, male gametes, immediately after natural ejaculation (or recovered from the epididymis in IVF protocols) are unable to fertilize oocytes and need to complete the capacitation within the genital tract (or in an artificial *in vitro* system). During capacitation, sperm membranes are known to undergo a complex reorganization, activated by a cAMP/PKA-dependent pathway by HCO_3_^–^ and Ca^2+^ which are present at relatively high concentrations in both tubal fluid (Coy et al., 2012; Suarez, 2016) and *in vitro* capacitating media (Boerke et al., 2013; Leahy and Gadella, 2015). As a result, the mammalian sperm Plasma Membrane (PM) changes its chemical-physical composition, becoming more fluid and acquiring the ability to fuse with the Outer Acrosome Membrane (OAM) when it makes contact with the Zona Pellucida (ZP) (the so called “fusogenicity”). This last event is made possible thanks to the rupture of the PM asymmetry, the so called “lipid scrambling,” in which cholesterol is exposed on outer leaflet of sperm head membrane (Leahy and Gadella, 2015). Thus, in presence of extracellular acceptors, cholesterol is removed from PM, the ratio cholesterol/phospholipids decreases, and the membrane fluidity and fusogenicity increases. Additionally, cholesterol has been associated with the formation of Detergent-Resistant plasma Membrane (DRM) microdomains, i.e., specialized areas that can be isolated by using detergents, such as 1% Triton X-100, at 4°C. Among these, only some of them contain caveolin-1, suggesting that at least two types of microdomains are present in mouse sperm, caveolin-1-containing microdomains and non-caveolin or lipid raft domains (Brown and Jacobson, 2001). Membrane rafts are defined as small, heterogeneous, highly dynamic domains that serve to compartmentalize cellular processes (Pike, 2006). A multiplicity of cellular functions have been associated with these microdomains, such as membrane trafficking, cellular signal transduction, viral entry and sperm fertilization (Mishra and Joshi, 2007; Santos and Preta, 2018).

Initially, we had to address some issues related to direct and indirect potential toxicity of GO on germplasm and to fully decipher the biochemical nature of its effect on membrane compartmentalization. Specific toxicological effects that likely lead to embryos more prone to present epigenetic failures (e.g., BHW syndrome in human field and large offspring syndrome in bovine) represent one of the problems associated to traditional IVF procedures because they are related to evident consequences on newborns (Rossignol et al., 2006; Soejima and Higashimoto, 2013). We also attempted to verify the mechanism of action behind the improved fertilization rates by establishing a series of IVF trials using mouse sperm in conjunction with contemporary IVF techniques.

The potential detrimental effects of graphene-based materials were suggested by recent findings reported by Asghar et al. (2016), who demonstrated that the presence of functional single walled carbon nanotubes (SWCNT-COOH) and reduced graphene oxide (rGO) at concentrations below 25 μg/ml did not affect sperm viability. In contrast, SWCNT-COOH (25 μg/ml) generated significant reactive superoxide species (ROS) (Asghar et al., 2016). We considered this last finding of great interest and worthy of further investigation, since spermatozoa exposure to high amounts of ROS could directly or indirectly affect sperm viability and DNA fragmentation (Kumaresan et al., 2017).

Our findings suggest that the exposure of mouse spermatozoa to GO, following a standard IVF protocol, did not affect the embryo survival and could increase the birth rate. Exploring GO concentrations ranging from 0.1 to 50 μg/ml, we found a complex dose-response between GO concentrations and IVF outcome. In particular, we found a positive effect of 0.1 and 0.5 μg/ml (i.e., 17.9 and 20.1 IVF% of increase, respectively), while higher concentrations show an increasing negative affect (e.g., highest GO concentration caused a −28.5 ΔIVF% decrease).

Then, we focused our analysis on 0.5 μg/ml GO and we did not detect any statistically difference in terms of early embryo development, while we found a statistically evident positive birth rate effect by implanting the embryos (IVF generated) from spermatozoa exposed to GO 0.5 μg/ml. Within a sperm sample, several subpopulations of spermatozoa coexist moment by moment, among which only a very small percentage of sperm cells are able to fertilize (less than 10%). In consequence, only when capacitating spermatozoa are seriously damaged the whole fertilizing ability is compromised. Naturally, a relevant percentage of non-fertilizing spermatozoa with impaired acrosomes does not affect IVF outcomes. Probably, in our system the GO action could exert its beneficial effects on IVF by increasing the number of capacitating spermatozoa, while impairments may involve other subpopulations.

We then carried out two different experiments in an attempt to establish how GO can enhance the sperm function. Firstly, we compared the effect derived from sperm exposure to GO with that exerted by MβCD on IVF outcomes. MβCD is a water-soluble oligosaccharide containing a hydrophobic cavity and characterized by α-(1-4) linked D-glycopyranose units able to form heptamers. It is characterized by high solubility in water, an ability to form a complex with aromatic or heterocyclic compounds, and a high aptitude to bind cholesterol, thus being a cholesterol acceptor widely used in murine IVF (Takeo et al., 2008; Takeo and Nakagata, 2010).

As evident from the data shown in Table IV, GO 0.5 μg/ml and MB CD 0.75 mM have a beneficial effect on IVF outcomes and, what is more, they seem to have a synergistic effect, leading us to the hypothesis that they may be exerting different chemical effects on sperm membranes.

Secondly, to study the effects of GO exposure on refurbishing the sperm surface architecture during spermatozoa incubation and under capacitating conditions, we examined a MEF, prepared from sperm incubated in a capacitating medium (BSA, MβCD, or GO). A number of different types of DRM microdomains have been described in the literature (Brown and Jacobson, 2001; Chamberlain, 2004). In this study we have used the general term TI (*triton insoluble* fraction) to describe membrane fractions which contain both *caveolae* and lipid rafts, and TS (*triton soluble* fraction) to describe all the rest of the membrane. A MEF was used as a starting material for isolation of TI. In detail, based on a previously published model (Bernabò et al., 2019), we compared control samples (i.e., without cholesterol acceptors) with GO treatment and with samples treated with bovine serum albumin (BSA), which is a physiological extracellular acceptor of cholesterol, and MβCD, a stronger cholesterol extractor routinely used in mouse IVF, as mentioned above.

We isolated the TI membrane fraction which contains both *caveolae* and lipid rafts. Consistent with our previous findings (Botto et al., 2010) we identified Cav-1 anomalous distribution since it was enriched in the TI fraction after BSA and GO treatment, but not after exposure to MβCD. This was not an artifact of the extraction procedure since membrane domain purification was confirmed by the presence of CD55 (marker of lipid rafts) only in the TI fractions. Then behavior of Cav-1, after BSA and GO treatment, suggests that this protein plays a relevant role during capacitation.

Shadan et al. (2004) showed that a low level of cholesterol efflux, mediated by MβCD, enhanced capacitation and induced the phosphorylation of capacitation-specific proteins. The cholesterol efflux associated with capacitation had no effect on the composition of the rafts, but destabilized them completely, also involving the suppression of the phosphorylation events. They concluded that there is a safe window for removing the sperm plasma membrane cholesterol. Outside this window, membrane disassembling becomes excessive, leading to disruption of lipid rafts. Hereby, it is possible to speculate that the localization of Cav-1 in the TI fraction after GO treatment (similar to that after BSA) could keep “the window open,” stabilizing the membrane domains. All these remodeling events associated with capacitation may facilitate the assembly of membrane insoluble microdomains and promote further recruitment of proteins, making membrane rafts an appropriate and indispensable remodeled platform to achieve a consistent fertilization rate. According to this interpretation, a relevant increase of the protein content was recorded in the TI fraction after carrying out all the capacitating treatments.

Cholesterol is generally recognized as a necessary component to maintain raft integrity; however, the treatment of spermatozoa with cholesterol acceptors does not compromise membrane raft composition (Shadan et al., 2004; Nixon et al., 2009). Our results support the hypothesis that BSA, MβCD, and GO preferentially extracts cholesterol from the external areas of the membrane rather than from the internal areas, identifying GO as the most effective cholesterol extractor discovered. Moreover, this study demonstrated how GO is able to modify the membrane fluidity without interfering with the sperm function, i.e., without destroying DRMs and is therefore a valuable alternative to the classical cholesterol acceptors already in use.

It could be considered of relevant interest elucidating the molecular mechanism on which the GO–sperm membranes interaction is based, providing germane information potentially useful to engineer and maximize the system. For instance, it could be possible to functionalize GO, controlling and increasing its effects and thus targeting very precisely its interaction with specific lipid or protein targets. However, this matter opens the way to a completely new line of research that can be the object of further research in our laboratory.

## Conclusion

GO was demonstrated to be an effective cholesterol extractor from the more fluid part of membrane surrounding DRMs (liquid disordered, L_D_) without affecting microdomains integrity (where the signaling systems are located) and showed an atypical chemical behavior. GO treatment, at a concentration of 0.5 μg/ml, improved IVF outcomes in laboratory mice, without affecting the early embryo development, increased birth rate and illustrated a synergic activity when used simultaneously with MβCD.

Taken together, these findings open new and interesting perspectives regarding the use of GO as a biomaterial for translational and personalized medicine, especially to be applied in the study and treatment of reproductive pathologies and infertility.

In addition, our experiments shed light on the interaction of a new material, GO, and cellular systems, thus providing information that could be useful for a wide spectrum of researches on biological field as well as for the research community devoted to materials science.

## Data Availability Statement

All datasets generated for this study are included in the article/Supplementary Material.

## Ethics Statement

The animal study was reviewed and approved by CNR-IBCN/Infrafrontier—Animal Welfare and Ethical Review Body (AWERB), in compliance with the Legislative Decree 26/2014 (ref. A80EE.N.7KR).

## Author Contributions

NB, LV, and MR: participation in study design. LB, LV, MR-S, JM-S, SA, FS, RP, and SP: execution. NB, AF, LB, PP, RP, LV, FS, and MF: analysis. NB, MR-S, LV, MR, AF, MF, and BB: manuscript drafting and critical discussion. All authors contributed to the manuscript and approved the submitted version.

## Conflict of Interest

The authors declare that the research was conducted in the absence of any commercial or financial relationships that could be construed as a potential conflict of interest.

## Footnotes

1 https://www.grandviewresearch.com/press-release/global-assisted-reproductive-technology-market

2 https://www.genome.gov/10001345/importance-of-mouse-genome

3 https://genome.cshlp.org/content/15/12/1729.full.html

4 https://www.infrafrontier.eu/

## Supplementary Material

DATA SHEET S1 IVF, ET, and birth rates data. The Excel file collects all the data regarding the number of animals, number of oocytes statistical analysis for IVF (first sheet) and embryo viability (second sheet).

DATA SHEET S2 Western Blotting and Red Ponceau staining. Red Ponceau staining and supporting Western Blotting images corresponding to the CD55 and CAV-1. The images are representative from three independent experiments.
