# Supplementary material for: Graphene Oxide Improves in vitro Fertilization in Mice With No Impact on Embryo Development and Preserves the Membrane Microdomains Architecture
Source: Front Bioeng Biotechnol. 2020 Jun 16;8:629. doi: 10.3389/fbioe.2020.00629 (PMC7308453; doi:10.3389/fbioe.2020.00629)

RED PONCEAU STAINING

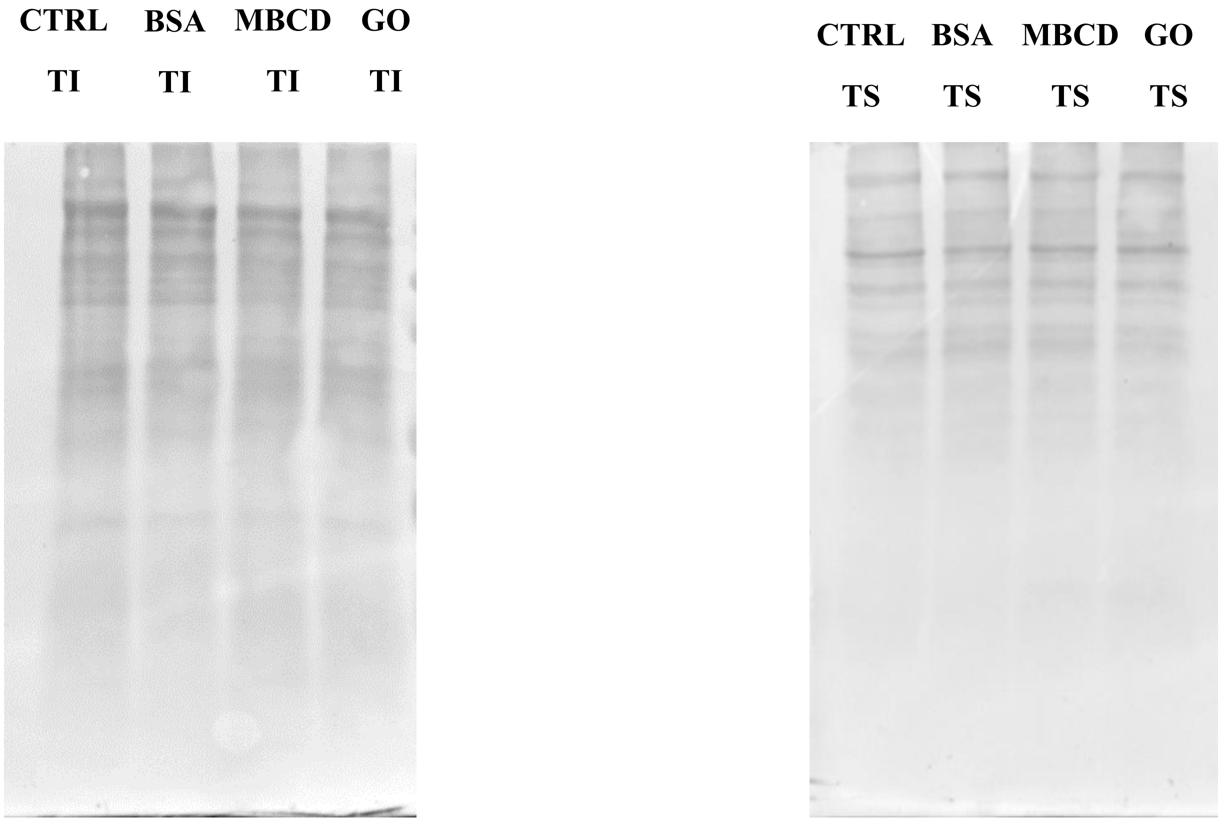

WESTERN BLOTTING IMAGES

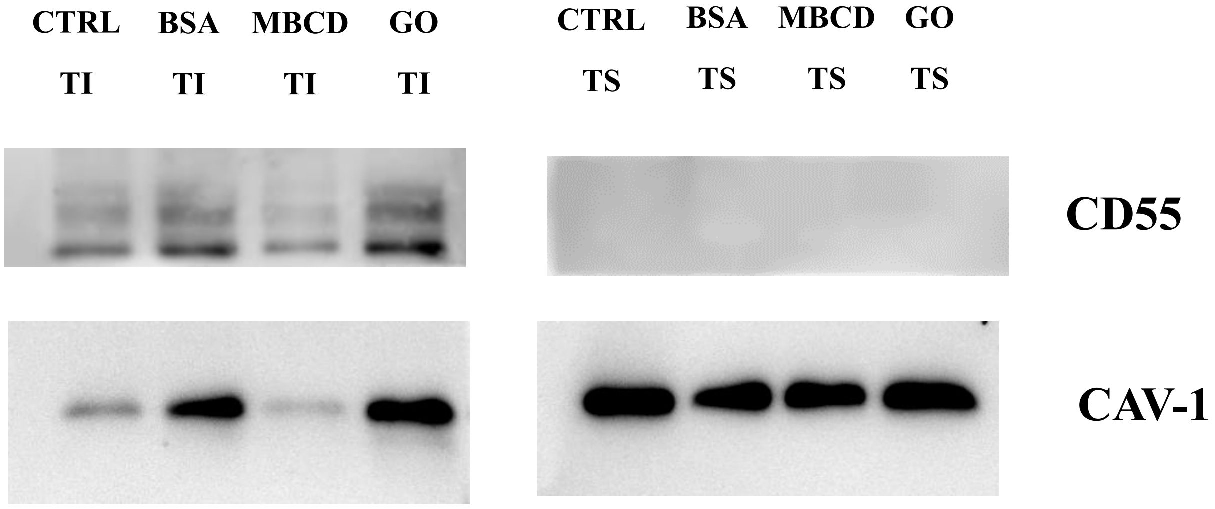

Supplement: DATA SHEET S2 — Western Blotting and Red Ponceau staining. Red Ponceau staining and supporting Western Blotting images corresponding to the CD55 and CAV-1. The images are representative from three independent experiments. [file Data_Sheet_2.pdf]
